# Facile fabrication of multi-pocket nanoparticles with stepwise size transition for promoting deep penetration and tumor targeting

**DOI:** 10.1186/s12951-021-00854-z

**Published:** 2021-04-19

**Authors:** Xingyu Hou, Dan Zhong, Yunkun Li, Hongli Mao, Jun Yang, Hu Zhang, Kui Luo, Qiyong Gong, Zhongwei Gu

**Affiliations:** 1 Huaxi MR Research Center (HMRRC), Department of Radiology, Functional and Molecular Imaging Key Laboratory of Sichuan Province, National Clinical Research Center for Geriatrics, West China Hospital, Sichuan University, Chengdu, 610041 People’s Republic of China; 2 Research Institute for Biomaterials, Tech Institute for Advanced Materials, College of Materials Science and Engineering, NJTech-BARTY Joint Research Center for Innovative Medical Technology, Nanjing Tech University, Nanjing, 211816 People’s Republic of China; 3 The Key Laboratory of Bioactive Materials, Ministry of Education, College of Life Science, Nankai University, Tianjin, 300071 People’s Republic of China; 4 Amgen Bioprocessing Centre, Keck Graduate Institute, Claremont, CA 91711 USA

**Keywords:** Drug delivery, Particle size, Tumor penetration, Disulfide cross-linking, Facile preparation

## Abstract

**Background:**

Nanocarriers-derived antitumor therapeutics are often associated with issues of limited tumor penetration and dissatisfactory antitumor efficacies. Some multistage delivery systems have been constructed to address these issues, but they are often accompanied with complicated manufacture processes and undesirable biocompatibility, which hinder their further application in clinical practices. Herein, a novel dual-responsive multi-pocket nanoparticle was conveniently constructed through self-assembly and cross-linking of amphiphilic methoxypolyethylene glycol-lipoic acid (mPEG-LA) conjugates to enhance tumor penetration and antitumor efficacy.

**Results:**

The multi-pocket nanoparticles (MPNs) had a relatively large size of ~ 170 nm at physiological pH which results in prolonged blood circulation and enhanced accumulation at the tumor site. But once extravasated into acidic tumor interstices, the increased solubility of PEG led to breakage of the supramolecular nanostructure and dissolution of MPNs to small-sized (< 20 nm) nanoparticles, promoting deep penetration and distribution in tumor tissues. Furthermore, MPNs exhibited not only an excellent stable nanostructure for antitumor doxorubicin (DOX) loading, but rapid dissociation of the nanostructure under an intracellular reductive environment. With the capacity of long blood circulation, deep tumor penetration and fast intracellular drug release, the DOX-loaded multi-pocket nanoparticles demonstrated superior antitumor activities against large 4T1 tumor (~ 250 mm^3^) bearing mice with reduced side effect.

**Conclusions:**

Our facile fabrication of multi-pocket nanoparticles provided a promising way in improving solid tumor penetration and achieving a great therapeutic efficacy.

**Graphic Abstract:**

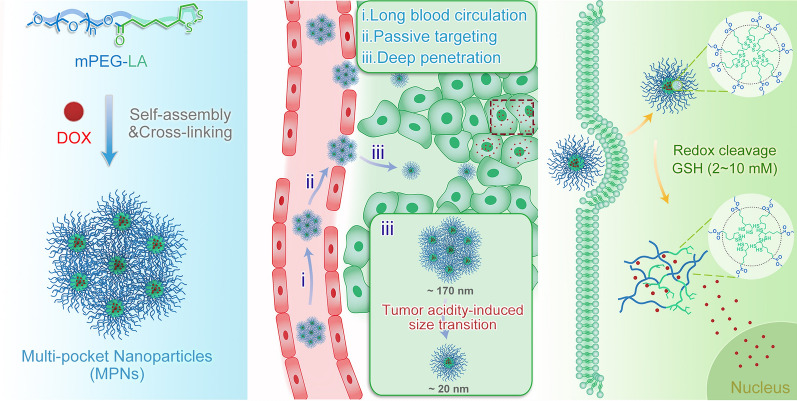

**Supplementary Information:**

The online version contains supplementary material available at 10.1186/s12951-021-00854-z.

## Introduction

Nanoscale supramolecular assemblies have been extensively exploited as drug carriers for cancer therapy over the last decades due to their improved drug solubility and pharmacokinetics, enhanced permeability and retention (EPR) at tumor site, and reduced systemic side effects [1–3]. Nevertheless, conventional self-assemblies formed via weak non-covalent hydrophobic interactions are inherently unstable in vivo because of their potential dynamic dissociation upon dilution, which often leads to premature drug release before reaching the tumor sites [4, 5]. Although some chemical approaches (e.g., cleavable covalent cross-linking via disulfide bonds [6–8], boronate bonds [9] and ketal bonds [10]) have been adopted to stabilize the assemblies and trigger site-specific drug release, features such as sophisticated nanostructures, biocompatibility and degradability, facile manufacture and low cost [11] are fundamental prerequisites for biomedical applications in antitumor drug delivery.

On the other hand, currently approved nanoscaled assemblies provide modest therapeutic efficacies, probably due to poor tumor penetration efficiencies resulted from abnormal tumor vasculature, elevated interstitial fluid pressure and dense interstitial matrix [12, 13]. Generally, large nanoparticles with a diameter of 70 ~ 200 nm are suitable for passive tumor accumulation via the EPR effect, but they have huge diffusional hindrance in the interstitial space [14]. On the contrary, small-sized nanoparticles (< 30 nm) have much better tumor penetration, while associated with a short half-life and poor tumor accumulation due to rapid elimination during circulation [14, 15]. Therefore, a size-switchable delivery system: a large size to achieve long blood circulation time and effective tumor accumulation before reaching the tumor site, but a small size for enhanced tumor penetration and uniform distribution once they are extravasated into tumor tissue, is pursued to improve therapeutic efficacies.

**Scheme 1:**
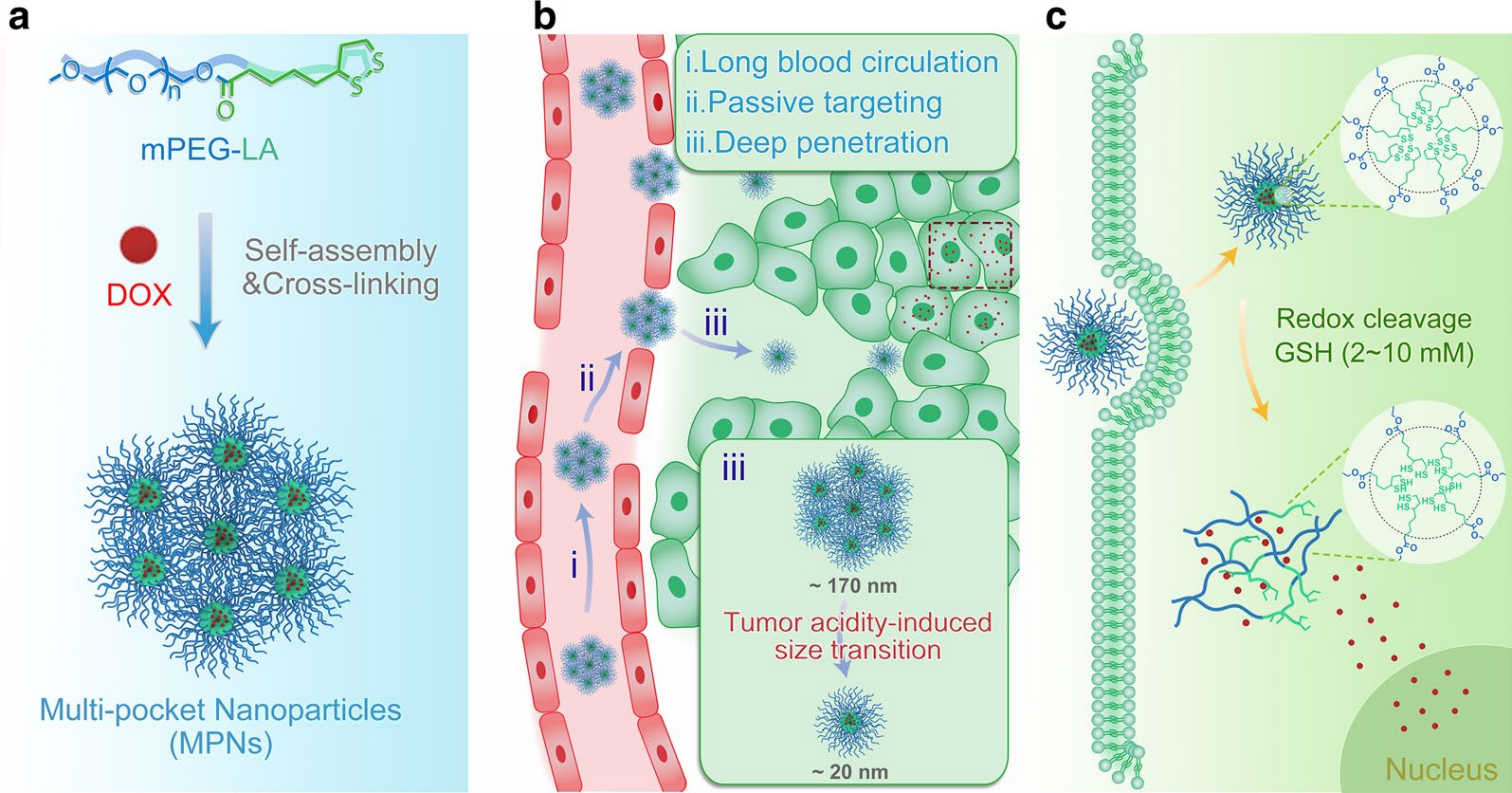
**a** Schematic illustration of construction of multi-pocket nanoparticles. **b** Elucidation of in vivo drug delivery of DOX-loaded MPNs. (i) A large supramolecular structure and PEGylation of MPNs prolong their blood circulation time; (ii) Prolonged circulation facilitates accumulation of large MPNs at tumor sites via the EPR effect; (iii) Shrinkage of MPNs into small-sized nanoparticles in tumor extracellular acidic environment is favourable for deep tumor penetration. (C) Cleavage of disulfide linkages and subsequent disintegration of small nanoparticles in an intracellular reductive environment lead to DOX release into cytoplasm and nucleus

Some size-switchable delivery systems that shrank their sizes in response of exogenous stimuli (e.g., light) or endogenous biological stimuli (e.g., adenosine triphosphate, enzymes, tumor acidity and reactive oxygen species) showed better tumor accumulation, deeper tumor penetration, and improved cancer treatment efficacy [16–20]. However, considering the difficulty of clinical translation, efforts should be made to reduce the complexity of the system [21]. Herein, we proposed a facile approach to fabricate novel multi-pocket nanoparticles (MPNs) which are size-manipulatable in the tumor acidic extracellular environment and structure-manipulatable to control the release of drug in the intracellular reductive environment (Scheme 1). The multi-pocket nanoparticles were readily prepared from methoxypolyethylene glycol-lipoic acid (mPEG-LA) conjugates through self-assembly and cross-linking in an aqueous solution. Polyethylene glycol (PEG) has been approved for clinical use by the US Food and Drug Administration (FDA) and it has been widely used in biomedical applications on account of its prolonged body-residence time, increased structure stability against proteases or nucleases, and reduction in immunogenicity [22, 23]. Lipoic acid is a natural product in mitochondria and it is usually used as an antioxidant drug for treatment of diabetes [24, 25]. At physiological pH during blood circulation, the prepared MPNs are expected to have excellent stability with a large size of ~ 170 nm, have a long blood circulation time and promoted tumor accumulation via the EPR effect. After reaching the tumor site, the tumor extracellular acidity (pH 6.5) activates the shrinkage of the supramolecular aggregates into small-sized disulfide cross-linked nanoparticles (< 20 nm) that enables deep tumor penetration to reach more cancer cells. Finally, intracellular glutathione (GSH) at a high concentration cleaves disulfide linkages to promote dissociation of cross-linked nanoparticles, and the drug is rapidly released to exert cytotoxicity against tumor cells.

## Results and discussion

### Preparation and characterization of MPNs

**Fig. 1:**
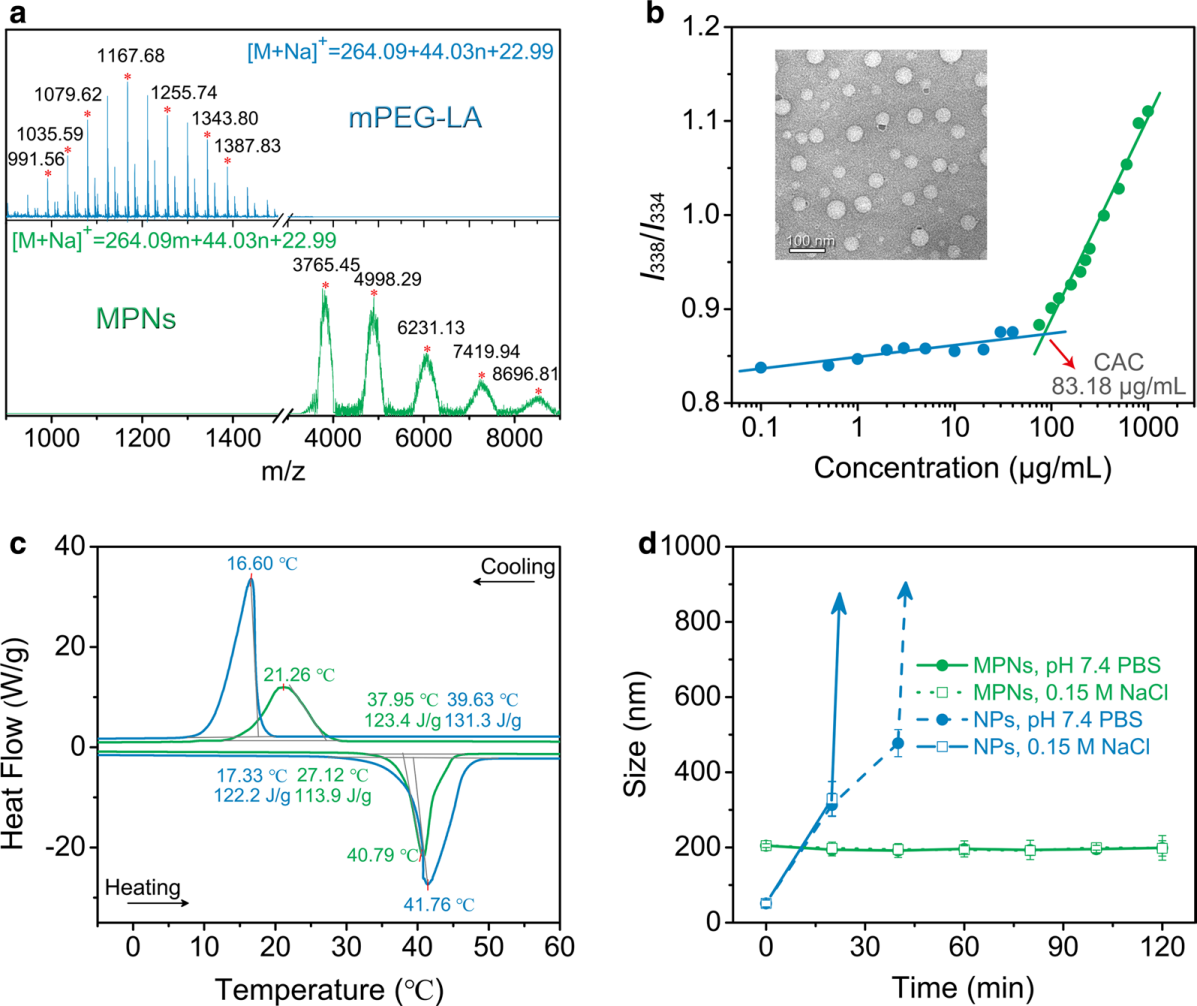
Characterization of NPs and MPNs. **a** MALDI-TOF mass spectra of mPEG-LA (blue) and MPNs (green). **b** Relationship between the fluorescence intensity ratio (*I*_338_/*I*_334_) and the mPEG-LA concentration in an aqueous solution at 25 ℃, [pyrene] = 6.7 × 10^–7^ M. Insert: transmission electron microscope (TEM) image of NPs. Scale bar: 100 nm. **c** DSC heating and cooling curves (10 ℃ min^−1^) for mPEG-LA (blue curve) and MPNs (green curve) from – 5 to 60 ℃ in nitrogen atmosphere. **d** Time-dependent size variation of NPs and MPNs at a physiological pH and a salt concentration at 25 ℃ (*n* = 3)

The amphiphilic mPEG-LA conjugates were readily prepared by treating mPEG with lipoic acid. ^1^H NMR spectrum in Additional file 1: Fig. S1 revealed that lipoic acid was successfully conjugated onto mPEG, and in the matrix-assisted laser desorption/ionization time-of-flight mass spectrum (MALDI-TOF MS) the peaks corresponded to the theoretical [M + Na]^+^ ions, where M = 264.09 + 44.03n and n = number of repeat units (Fig. 1a). The amphiphilic mPEG-LA self-assembled into uniform-nanosized spheres in aqueous medium with an average size of 40 nm and a negative surface charge of -18.1 mV (Additional file 1: Fig. S2). The critical assembly concentration (CAC) of the mPEG-LA conjugates using pyrene as a fluorescence probe was determined to be 83.18 μg mL^−1^ (Fig. 1b), and this quite high CAC suggested that they may be subjected to dissociate during blood circulation. After cross-linking under catalysis by DTT (10 mol% relative to the lipoyl units), the mass spectrum exhibited an increased molecular weight with several distributions, and the mass difference between two adjacent distributions equalled to the average molecular weight of the mPEG-LA conjugates, as a consequence of the formation of linear disulfide bonds between lipoyl units (Fig. 1a). Meanwhile, differential scanning calorimetry (DSC) curves revealed a lower melting temperature and a higher crystallization temperature of MPNs than pure mPEG-LA conjugates (Fig. 1c). The difference may be explained by that the cross-linking of disulfides greatly restricted the diffusion of molten polymer chains participating in the crystallization [26], leading to a reduction in the crystallinity. Dynamic light scattering (DLS) results confirmed that MPNs were stable against a physiological salt concentration (0.15 M NaCl) as well as a physiological pH (pH 7.4): the average size of MPNs remained unchanged (Fig. 1d), while the size of the non-cross-linked self-assemblies of mPEG-LA (Nanoparticles, NPs) gradually became undetectable or large aggregates were formed. Upon dilution to a low concentration of 10 μg mL^−1^, the size of MPNs still kept at about 170 nm, indicating a good stability and dispersity (Additional file 1: Fig. S3).

### Redox sensitivity of MPNs and controlled dye release

**Fig. 2:**
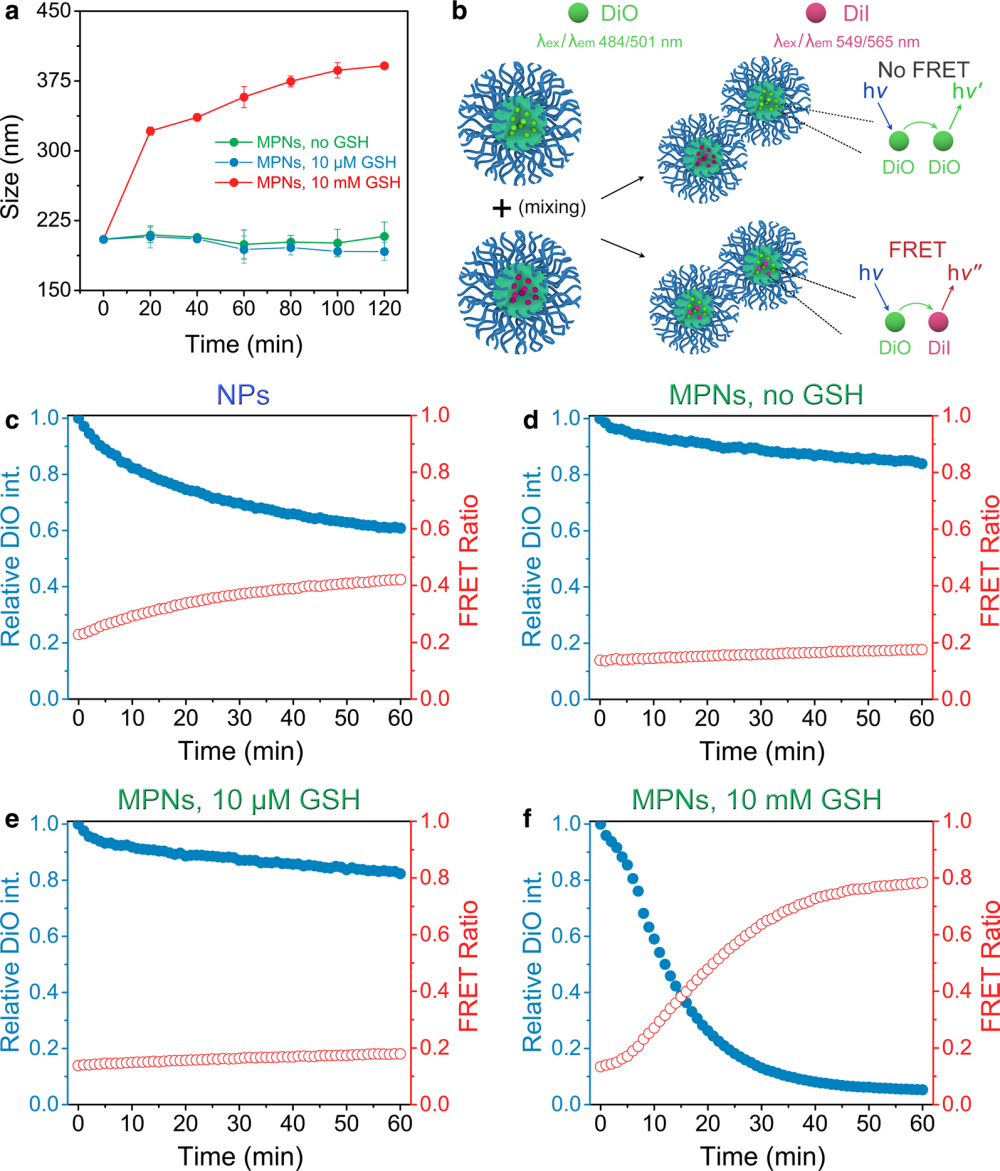
Redox responsiveness of MPNs. **a** Time-dependent size changes of MPNs in response to different concentrations of GSH measured by DLS at 25 ℃ (*n* = 3). **b** Schematic presentation of the potential FRET outcomes. **c**, **d**, **e**, **f** Time-dependent normalized (Norm.) emission intensity (int.) of DiO (blue solid dots, λex = 450 nm, λem = 510 nm) and change in FRET ratio (red circles) for NPs and MPNs with GSH

The redox responsiveness of MPNs was assessed by detecting changes in the size over time in a reductive environment similar to that in the extracellular matrix or in the cytoplasm. After they were exposed to 10 mM GSH corresponding to an intracellular reductive condition, the size of MPNs rapidly expanded to about 400 nm as a result of cleavage of disulfide linkages and disassociation of PEG chains (Fig. 2a). In addition, MPNs maintained stable in the buffer with 10 μM GSH analogous to an extracellular reductive environment. Subsequently, förster resonance energy transfer (FRET) experiment was employed to assess the encapsulation stability of MPNs [27]. The FRET pair dye consisting of lipophilic DiO (donor) and DiI (acceptor) was separately encapsulated in MPNs and NPs. If the dye molecules were stably trapped in the nanoparticles interior, no FRET would occur. But if the dye molecules were released from the nanoparticles and were within the förster radius, the DiO emission intensity decreased with development of FRET (Fig. 2b). When the DiO-containing NPs solution was mixed with the DiI-containing NPs solution, the DiO intensity rapidly decreased over time and the FRET ratio *I*_a_/(*I*_d_ + *I*_a_), where *I*_a_ and *I*_d_ were the fluorescence intensities of DiO and DiI, correspondingly increased (Fig. 2c and Additional file 1: Fig. S4). Notably, a much slower rate of FRET development was found in the solution of MPNs than that of NPs (Fig. 2d), confirming that disulfide cross-linking increased the encapsulation stability of the MPNs. The addition of 10 mM GSH to the MPNs solution immediately triggered fast release of the loading dyes (Fig. 2f), while the dye release was not affected in the presence of 10 μM GSH (Fig. 2e). The above results indicated that lipophilic molecules could be stably encapsulated in the MPNs during systemic circulation, but they could be rapidly released in an intracellular reductive environment after endocytosis.

### Tumor acidity-triggered size transition of MPNs

**Fig. 3:**
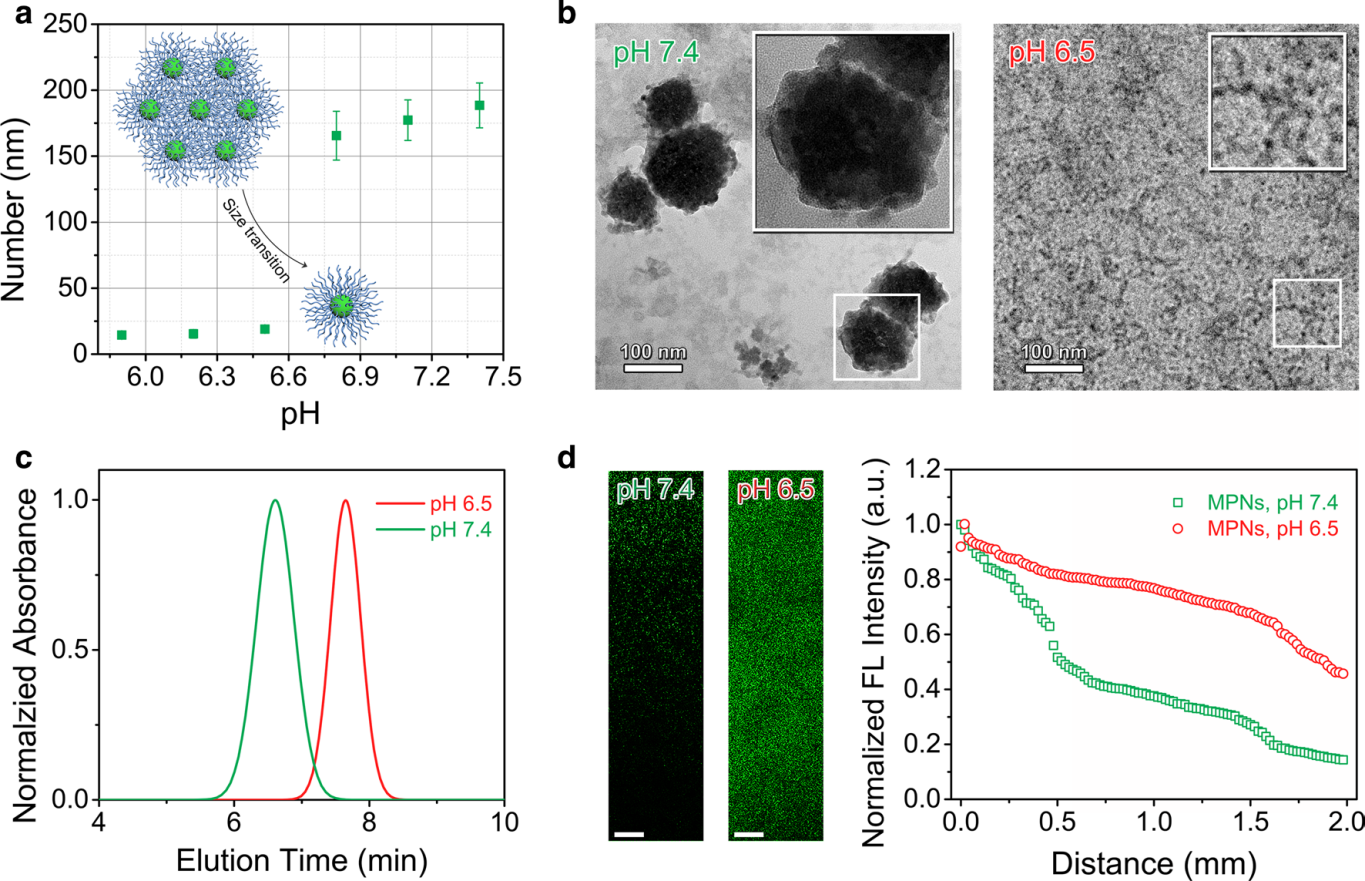
Tumor acidity-triggered size transition of MPNs. **a** Particle size of MPNs at different pH values. **b** TEM measurements of MPNs treated at pH 7.4 and 6.5. Insert: high magnification TEM image of MPNs. The NPs and MPNs were stained with 2 wt % phosphotungstic acid. **c** Gel permeation chromatography analysis of MPNs treated at pH 7.4 and 6.5. **d** Penetration profile of FITC-labeled MPNs (left panel) into the collagen hydrogel and normalized fluorescent intensity (right panel) of FITC-labeled MPNs in collagen hydrogels at pH 7.4 and 6.5. Scale bars: 125 μm

To evaluate the pH-sensitivity of MPNs, DLS was applied to measure their size change in water of different pHs ranging from 5.9 to 7.4. As shown in Fig. 3a, the size of MPNs varied from ~ 170 nm at pH 7.4 down to ~ 20 nm at pH 6.5. Therefore, we speculated that at pH 7.4, cross-linked nanoparticles were susceptible to aggregating into large nanostructures because of inter-micellar interactions through PEG corona [28], whereas at pH 6.5, the increased solubility of PEG in water and decreased inter-connection of PEG chains caused by the more effective hydrogen bonding of hydronium ions (H_3_O^+^) than water (H_2_O) to ethylene glycol units might lead to breakage of the supramolecular nanostructure and dissolution to individual cross-linked nanoparticles [29, 30]. The particle size of the core cross-linked nanoparticles was smaller than that of NPs, which might be attributed to the more compact nanostructure of the core cross-linked nanoparticles after cross-linking of inner disulfide bonds [31, 32]. The measured critical aggregation concentration of MPNs at pH 7.4 (25.23 μg mL^−1^) was 2.6-fold lower than that at pH 6.5 (64.57 μg mL^−1^, Additional file 1: Fig. S5). The morphology of MPNs viewed under a TEM directly evidenced that supramolecular aggregates appeared at pH 7.4, but small nanoparticles around 10 nm at pH 6.5 (Fig. 3b), suggesting acid pH-induced size change occurred for MPNs. Additionally, in the gel permeation chromatography (GPC) MPNs had a longer retention time at pH 6.5 than those at pH 7.4 (Fig. 3c), which further confirmed acid-activated dissolution of the superstructure into small particles. By the way, the dissolution of MPNs at pH 6.5 did not accelerate the release of encapsulated dyes (Additional file 1: Fig. S6).

In light of a small size of MPNs at the tumoral acidity (pH 6.5), it was reasonable to expect deep penetration of MPNs in tumor tissues. The diffusive transport of MPNs was evaluated using a collagen gel to stimulate the interstitial matrix of solid tumors in vitro. After pre-treatment at pH 7.4 or 6.5 for 1 h, 10 μL of FITC-labelled MPNs was placed in contact with the collagen gel and incubated at 37 ℃ for another 12 h. Confocal laser scanning microscopy (CLSM) was employed to gain an insight into the infiltration of both samples into the collagen gel (Fig. 3d). MPNs incubated at pH 7.4 displayed limited penetration, while MPNs incubated at pH 6.5 were able to penetrate a millimeter deep into the gel as a result of the dissolution of MPNs.

### Drug loading and release

**Fig. 4:**
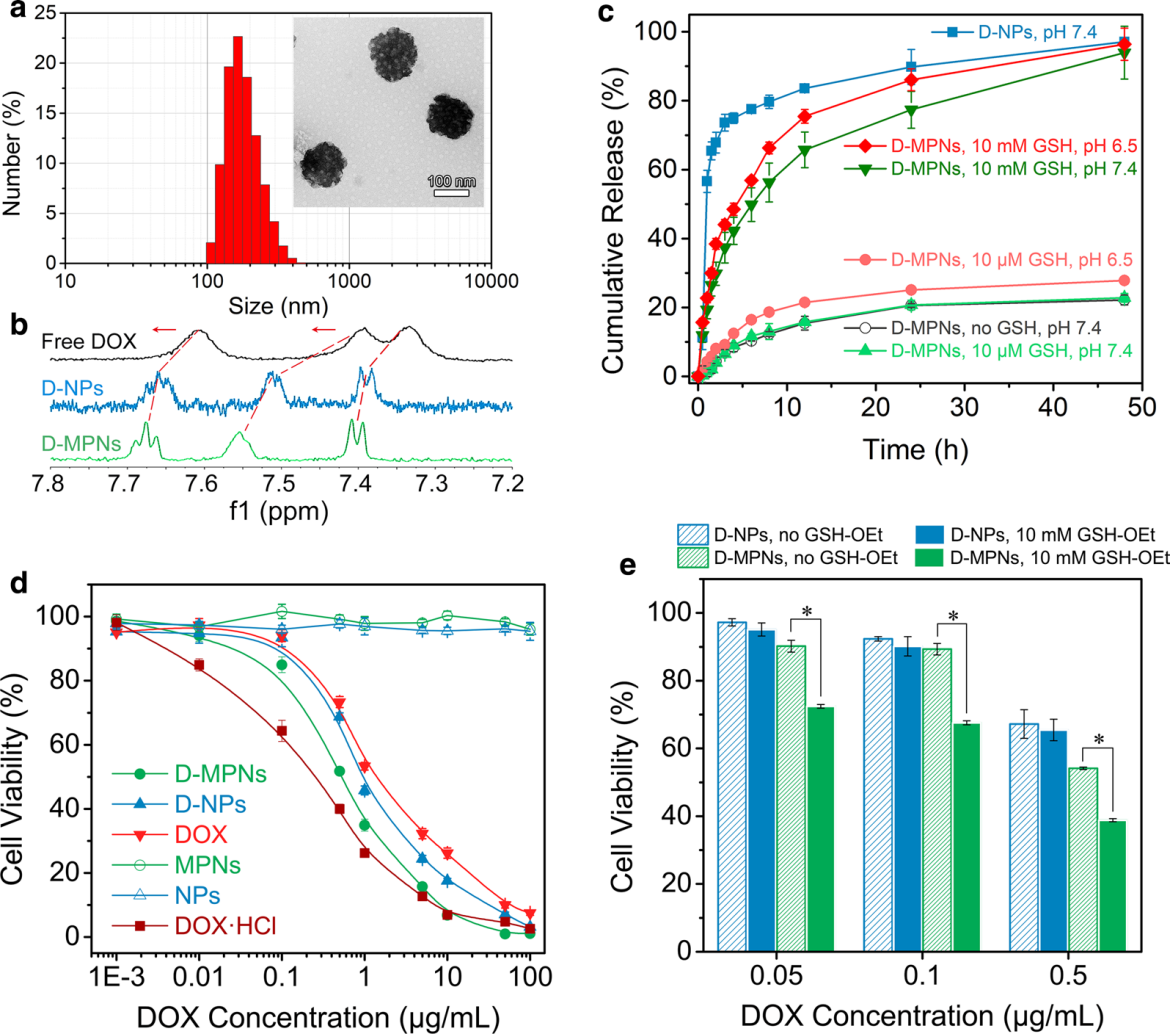
**a** Size distribution and TEM image (the insert) of D-MPNs. Scale bar: 100 nm. **b** Expansions of the ^1^H NMR spectra (600 MHz, D_2_O) of free DOX (top), D-NPs (middle) and D-MPNs (bottom). **c** Release of DOX from D-MPNs (100 μg mL^−1^) at different conditions mimicking physiological environments (means ± SD, *n* = 3). **d** Viabilities of 4T1 cells after treatment with NPs, MPNs, DOX, D-NPs and D-MPNs for 48 h (means ± SD, *n* = 5). **e** Proliferation inhibition of 4T1 cells incubated with D-NPs and D-MPNs at various DOX concentrations for 48 h. The cells were pretreated with 10 mM GSH-OEt for 2 h. The cells non-pretreated with GSH-OEt were used as control, (*p < 0.05)

In this work, hydrophobic doxorubicin (DOX) was used as a model drug and encapsulated into the nanoparticles to obtain DOX-loaded MPNs (D-MPNs) with a drug loading content of 11.94 wt%. DLS and TEM results indicated that D-MPNs displayed similar well-defined supramolecular nanostructures as original MPNs with a diameter of about 160 nm (Fig. 4a). ^1^H NMR spectra of free DOX, DOX-loaded nanoparticles (D-NPs) and D-MPNs also revealed some changes in the local environment of protons. A distinct change in the shape and position of DOX aromatic peaks was found after DOX was complexed with NPs or MPNs (Fig. 4b), indicating interactions among these chemical species [33]. The peaks of free DOX molecules at 7.33, 7.39 and 7.60 ppm were shifted downfield after complexation with NPs and MPNs, and the peaks of D-MPNs had more significant chemical shifts than those of D-NPs. Moreover, the signal-to-noise ratio of DOX when complexed with MPNs was significantly improved in comparison with that from D-NPs, which could be attributed to much more increased solubility of DOX in MPNs upon complexation.

The in vitro DOX release from D-MPNs under various biomimetic conditions were determined at a low concentration of 100 μg mL^−1^. As seen in Fig. 4c, burst release of DOX from D-NPs was observed under a normal physiological condition (pH 7.4), with the cumulative release up to 60% within 2 h, while a very slow DOX release from D-MPNs was observed in the same environment, due to their extremely stable nanostructure. At pH 6.5 with 10 μM GSH mimicking a tumor extracellular microenvironment, the DOX release rate was quite similar to that at pH 7.4. As expected, the intracellular reductive condition (10 mM GSH) triggered rapid DOX release from D-MPNs, and about 80% DOX was released within 24 h.

### In vitro antitumor activity

The in vitro antitumor activity of D-MPNs against mouse breast 4T1 tumor cells was determined by CCK-8 assays. The MPNs up to a high concentration of 1 mg mL^−1^ were nontoxic to 4T1 cells, while D-MPNs exhibited much stronger antitumor effects against 4T1 cells in comparison with D-NPs and DOX (Fig. 4d). The IC_50_ value (the concentration for inhibiting 50% of cell growth) for D-MPNs to 4T1 cells was 0.55 μg mL^−1^, which was lower than that of D-NPs (0.91 μg mL^−1^) and DOX (1.63 μg mL^−1^), this could be attributed to an improving bioavailability of the poorly soluble DOX by D-MPNs. Given the excellent antitumor effect of D-MPNs, a fluorescence-activated cell sorter (FACS) was utilized to assess drug internalization of D-MPNs. After exposure to different formulations for 3 h, the stronger fluorescence intensity was detected in D-MPN-treated cells (Additional file 1: Fig. S7), supporting that MPNs could efficiently promote cellular uptake of the antitumor drug DOX. Since DOX played its therapeutic role in the nuclei, delivery of D-MPNs into the nuclei was further confirmed by Hoechst 33,342 staining (Additional file 1: Fig. S8). It was revealed from the CLSM images that after incubation for 3 h, more DOX was delivered by MPNs into 4T1 cells and the nuclei to exert its antitumor activity. To elucidate the role of bioreducibility in drug delivery through MPNs, 4T1 cells were pretreated with glutathione monoester (GSH-OEt) to up-regulate the intracellular GSH level and followed by incubation with D-MPNs [34]. Enhanced inhibition activity of D-MPNs was observed with GSH-OEt pretreated 4T1 cells (Fig. 4e), since a high level of GSH could facilitate the breakage of cross-linked nanostructure and greatly accelerate the release of antitumor drugs to inhibit the tumor cell proliferation.

### Penetration in MTSs and tumor tissues

**Fig. 5:**
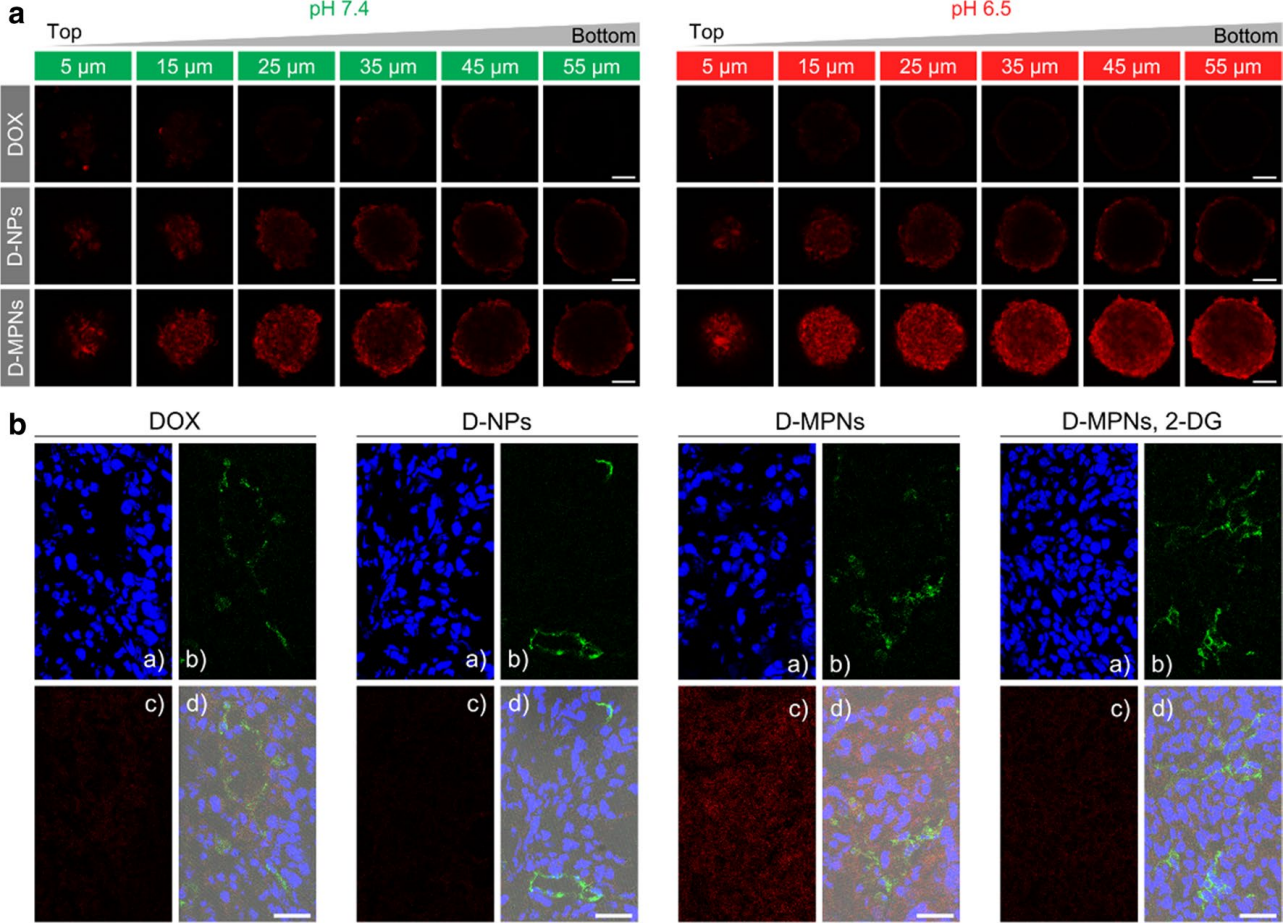
In vitro and in vivo drug penetration D-MPNs. **a** CLSM images of 4T1 multicellular tumor sections at a distance of 5, 15, 25, 35, 45 and 55 μm from the spheroid rim. The MTSs were incubated with DOX, D-NPs and D-MPNs in the culture media at pH 7.4 or 6.5 for 2 h (DOX concentrations: 5 μg mL^−1^). Scale bars: 50 μm. **b** CLSM image of 4T1 tumor sections after administration with DOX, D-NPs and D-MPNs for 12 h at a DOX dosage of 10 mg kg^−1^. Nuclei were stained by Hoechst 33,342 (**a**, blue) and blood vessels by FITC-CD31 (**b**, green). DOX fluorescence was visualized in red (**c**) and the merged imaged in (**d**). Scale bars: 30 μm

The tumor penetration efficacy of MPNs in the tumor tissue was evaluated through multicellular tumor spheroids (MTSs) with a diameter of about 200 μm as an in vitro tumor model. It has been demonstrated that MTSs could be served as a realistic and suitable three-dimensional model of solid tumors for investigating tumor biology and screening anticancer drugs by mimicking the complicated multicellular architecture and the biological microenvironment of solid tumors [35]. 4T1 MTSs were incubated with DOX, D-NPs or D-MPNs for 3 h at pH 7.4 and 6.5, respectively. Low-intensity fluorescence signals were seen on each equatorial section of 4T1 MTSs after treatment with DOX (Fig. 5a), due to intrinsic poor distribution of hydrophobic drugs. Meanwhile, D-MPNs showed a better penetration behavior than D-NPs at either pH 7.4 or 6.5, which may be ascribed to the disulfide cross-linked nanostructure of MPNs. There was no obvious difference in distribution of DOX or D-NPs in MTSs at both pH values. But excitingly, the penetration ability of D-MPNs at a pH of 6.5 was remarkably improved, and the percentage of DOX-positive cells in MTSs increased from 39.0% at pH 7.4 to 67.1% at pH 6.5 which was determined from FACS analysis (Additional file 1: Fig. S9), The enhanced drug penetration ability of D-MPNs may be due to acidic pH-stimulated rapid shrinkage of MPNs into small-sized nanoparticles.

The intratumoral drug distribution was further evaluated via cryosection of tumor tissues from mice bearing 4T1 tumors. From the CLSM images, it was seen that the red fluorescence intensity from DOX·HCl and D-NPs was quite weak around the FITC-anti-CD31-stained blood vessels, while the fluorescent intensity from D-MPNs was significantly enhanced and the signals spread much farther away from the tumor blood vessels (Fig. 5b). It is known that solid tumors have an acidic intratumoral microenvironment generated by an aerobic glycolysis [36]. To further confirm tumor acidity-activated penetration of D-MPNs in vivo, 2-deoxy-D-glucose (2-DG), a glycolytic inhibitor, was intratumorally injected to suppress uptake of glucose by tumor cells and prevent acidification in the tumoral microenvironment [37]. As shown in Fig. 5b, in the presence of 2-DG the penetration ability of D-MPNs significantly reduced as evidenced with attenuated fluorescence signals. The reduced penetration may be explained by inhibited acidification of the intratumoral environment and subsequent size decrease of MPNs. These results supported that intratumoral penetration of MPNs was tumor acidity-dependent in the in vitro MTSs model and in vivo mouse model.

### Pharmacokinetics, biodistribution and in vivo antitumor activity

**Fig. 6:**
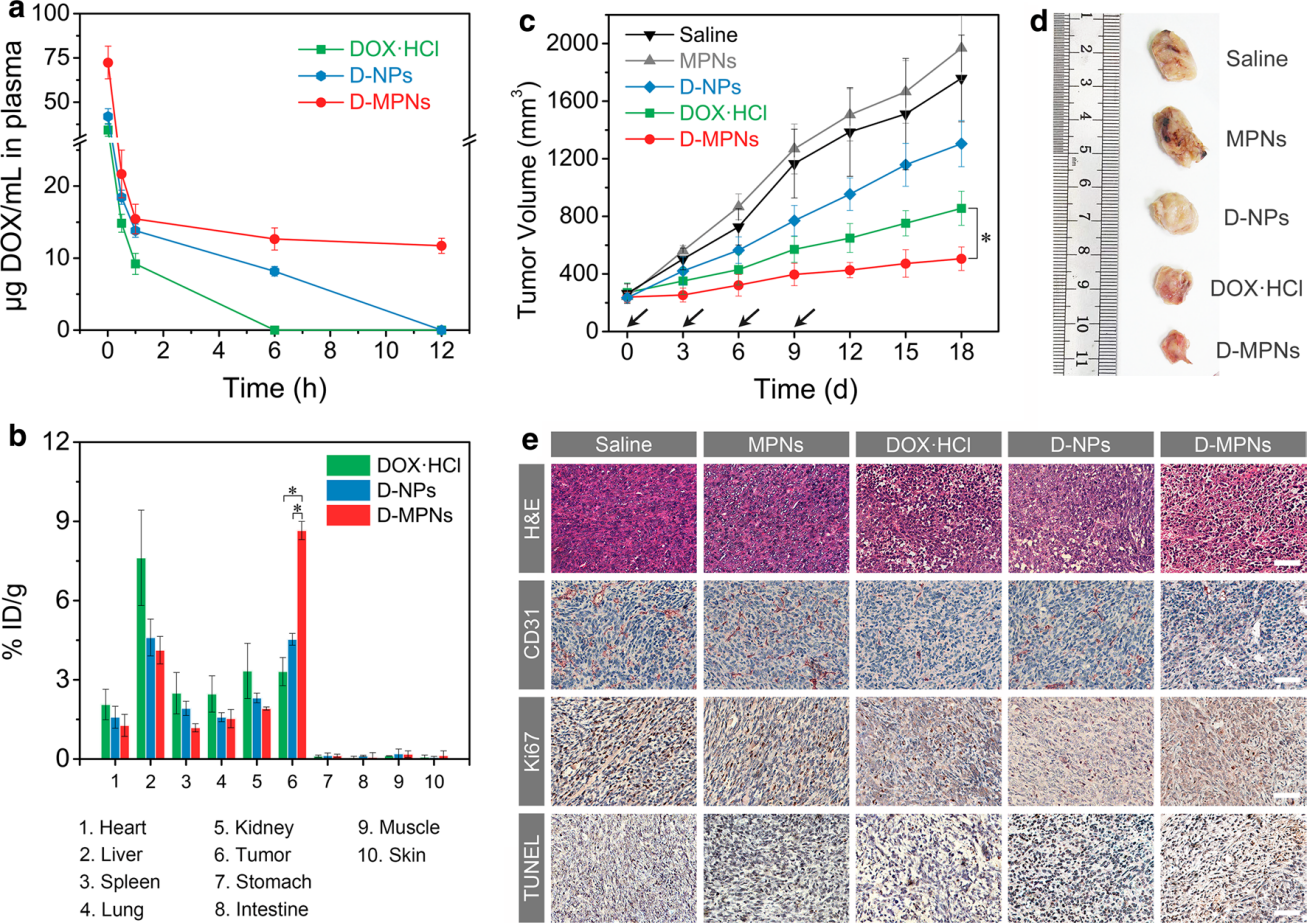
Evaluation of in vivo antitumor efficacy. **a** In vivo pharmacokinetics of DOX·HCl, D-NPs or D-MPNs (*n* = 3). **b** Biodistribution of DOX·HCl, D-NPs and D-MPNs in mice at 12 h post-injection. **c** Tumor volumes of 4T1-bearing mice after intravenous administration with saline, MPNs, DOX·HCl, D-NPs and D-MPNs at a DOX dosage of 5 mg kg^−1^ (*n* = 8, *p < 0.01). The arrows indicate the times of intravenous tail vein performance. **d** Photographs of typical tumors collected on day 18. **e** Immunohistochemistry staining of 4T1 tumors. CD31-positive microvessels, Ki67-positive cells and TUNEL-positive cells were stained brown. Scale bars: 50 μm

With a PEGylated corona and a disulfide cross-linked stable nanostructure, D-MPNs was expected to have prolonged systemic circulation in vivo. The pharmacokinetics studies was conducted with normal mice following intravenous administration of DOX·HCl, D-NPs and D-MPNs at various time points and the DOX content in plasma was analyzed by HPLC. The temporal changes in the drug concentration (Fig. 6a) and the pharmacokinetic parameters (Additional file 1: Table S1) calculated by non-compartmental analysis revealed that MPNs remarkably increased the area under the curve (AUC) for DOX in blood, which was 6.2-fold greater than that for D-NPs and 10.8-fold for DOX·HCl. Meanwhile, the mean retention time (MRT) of D-MPNs was 12.9-fold and 80.5-fold of D-NPs and DOX·HCl, respectively. In addition, the half-life period (t_1/2_) of D-MPNs (18.09 h) was much longer than that of D-NPs (1.81 h) and DOX·HCl (0.83 h).

The biodistribution of DOX in tumors and major organs at 12 h post-injection quantified by HPLC demonstrated that the mice treated with D-MPNs had a remarkably drug accumulation at the solid tumors of 8.6% ID g^−1^ (injected dose per gram of tissues), which was about 1.9 fold higher than that of D-NPs group and 2.6 fold higher than that of DOX·HCl group, respectively (Fig. 6b). Of note, the tumor accumulation of D-MPNs was also significantly higher than in the healthy organs such as heart, spleen, lung, and kidney.

Encouraged by the long blood circulation, enhanced tumor accumulation and deep tumor penetration, we evaluated the in vivo antitumor efficacy of D-MPNs on BALB/c mice bearing large 4T1 xenograft tumors (average size 250 mm^3^) following intravenous injection for 4 times. As presented in Fig. 6c, the tumor volumes of saline group increased to about 1800 mm^3^ after treatment over 18 d. And the treatment of D-NPs resulted in slight suppression of tumor growth due to an unstable nanostructure and immature drug release. Encouraging, D-MPNs showed remarkable tumor inhibition capacity on tumor growth, which was better than the DOX·HCl-treated positive control group (*p < 0.01). The tumors collected from mice treated with D-MPNs on day 18 was the smallest among all groups, which further confirmed the excellent antitumor efficacy of D-MPNs (Fig. 6d). Furthermore, hematoxylin and eosin (H&E) staining of tumor slices manifested that the treatment by D-MPNs led to remarkable damage to tumor cells in the tumor tissue evidenced by a loose structure and distinct karyopyknosis, whereas abundant and compact tumor cells were observed in the saline- and MPNs-treated groups (Fig. 6e). CD31-positive vessels were effectively reduced after administration with D-MPNs. More importantly, the fewest Ki67-positive proliferating cells as well as the most TUNEL-positive apoptotic cells were detected in the tumor tissues after D-MPNs treatment (Additional file 1: Fig. S10).

Favorable biocompatibility is crucial for potential clinical application of delivery systems. And body weight change acts as a common indicator for systemic toxicity assessment. After intravenous injection, the mice treated with D-MPNs showed a slight increase of body weight, while DOX·HCl caused a severe weight loss of ~ 7% (Additional file 1: Fig. S11). From blood routine analysis data, no differences in white blood cell (WBC), red blood cell (RBC), platelet (PLT) and hemoglobin (HGB) levels were observed in D-MPNs-treated group and saline-treated control group (Additional file 1: Fig. S12). No histopathological abnormality was found in H&E-stained sections of major organs in D-MPNs-treated group (Additional file 1: Fig. S13). In contrast, apoptotic bodies were found in heart tissues of DOX·HCl group by TUNEL staining (Additional file 1: Fig. S14). In general, these results demonstrated the excellent therapeutic efficiency and biocompatibility of D-MPNs, that was worthy for further research as a potential clinical antitumor agent.

## Conclusions

In conclusion, we have successfully fabricated a novel dual-responsive multi-pocket nanoparticles derived from self-assembly and cross-linking of amphiphilic mPEG-LA conjugates. The multi-pocket nanoparticles exhibited a long circulation time and tumor pH-triggered size reduction into smaller nanoparticles, which facilitated deep penetration into the tumor tissue. Moreover, the multi-pocket nanoparticles offered stable nanostructures for drug encapsulation, while the intracellular reductive environment cleaved disulfide linkages and triggered rapid drug release. The DOX-loaded multi-pocket nanoparticles showed superior antitumor activities against large 4T1 tumor (~ 250 mm^3^) bearing BALB/c mice with reduced side effects. Our facile fabrication of multi-pocket nanoparticles would provide a promising way in improving solid tumor penetration and achieving a great therapeutic efficacy.

## Experimental section

### Synthesis of mPEG-LA conjugates

mPEG (3.00 g, Mw 1000), lipoic acid (0.93 g, 4.50 mmol), EDC·HCl (0.86 g, 4.50 mmol) and HOBT (0.61 g, 4.50 mmol) were dissolved in dichloromethane (~ 50 mL) under nitrogen atmosphere. After cooled at 0 ℃ for 30 min, DIPEA (2.0 mL, 12.00 mmol) was added dropwise into the mixture. After reaction at room temperature for 12 h, the mixed solution was washed with saturated NaHCO_3_ and NaCl solution 3 times and dried with MgSO_4_ overnight. The crude product was purified by column chromatography using dichloromethane: methanol (v:v = 50:1) as an eluent. ^1^H NMR (400 MHz, CDCl_3_): δ = 4.26–4.19 (t, C*H*_*2*_OCO), 3.65 (m, C*H*_*2*_C*H*_*2*_O), 3.58–3.53 (m, SSCH_2_CH_2_C*H*), 3.38 (s, C*H*_*3*_O), 3.22–3.07 (m, SSC*H*_*2*_CH_2_CH), 2.46 (SSCH_2_C*H*_*2*_CH), 2.35 (t, C*H*_*2*_COO), 1.91 (m, SSCH_2_C*H*_*2*_CH), 1.76–1.59 (m, C*H*_*2*_CH_2_C*H*_*2*_CHS), 1.51 (m, CH_2_C*H*_*2*_CH_2_CHS).

### Self-assembly of mPEG-LA conjugates

Pyrene was used as a hydrophobic fluorescent probe to detect the critical assembly concentration (CAC) of mPEG-LA conjugates. Briefly, the concentration of pyrene was fixed at 0.67 μM and the concentrations of mPEG-LA varied from 0.1 to 1000 μg mL^−1^. The excitation spectra of pyrene were recorded from 300 to 380 nm using a fluorescence spectrometer (Hitachi F-7000) with an emission wavelength of 395 nm. The CAC was estimated according to the intersection of the curves when extrapolating the intensity ratio *I*_338_/*I*_334_ at low and high concentration regions.

### Preparation of MPNs and drug loaded MPNs

MPNs were prepared by adding a catalytic amount of DTT (10 mol% to lipoyl units) into mPEG-LA solution under a nitrogen atmosphere. After reaction for 24 h, the solution was dialyzed against water in a dialysis tube (Spectra/Por, MWCO 2000) and lyophilized. DOX was loaded into MPNs by adding a mixed solution of mPEG-LA conjugates (15 mg) and DOX (3 mg) in DMSO into 30 mL of water containing catalytic amount of DTT under an ultrasound condition. After 24 h, the solution was transferred to a dialysis tube (Spectra/Por MWCO 2000) and dialyzed against distilled water. One day later, the supernatant were lyophilized and the drug loading content (DLC) was determined by fluorescence measurement (excitation at 480 nm and emission at 550 nm).

### In vitro drug release

The release of DOX from DOX-loaded nanoparticles (D-NPs) and D-MPNs was studied in phosphate buffer (10 mM, pH 7.4 or pH 6.5) with or without GSH (10 μM or 10 mM). D-MPNs and D-NPs were prepared at a polymer concentration of 100 μg mL^−1^ (close to CAC) and transferred into dialysis tubes (MWCO 1000). The tubes were suspended in 25 mL of media. The samples were shaken at 120 rpm and 37 ℃. At different intervals, 1 mL of the medium in the tube was taken away for fluorescence measurement and 1 mL of fresh medium was supplemented. The amount of DOX released was quantified by a fluorescence spectrometer (excitation at 480 nm and emission at 550 nm).

### FRET measurements

A solution of MPNs encapsulating DiI (0.1 wt%, 500 µL of 0.2 mg mL^−1^ stock) was added to a solution of MPNs encapsulating DiO (0.1 wt%, 500 µL of 0.2 mg mL^−1^ stock). The fluorescence spectra over time were recorded at an excitation wavelength of 450 nm. And the FRET ratios were obtained from the equation *I*_a_/(*I*_d_ + *I*_a_), where *I*_a_ and *I*_d_ were the fluorescence intensities at 570 nm and 510 nm, respectively.

### Collagen gel diffusion

Collagen hydrogels were prepared by mixing 141.75 μL of rat tail collagen type I (5 mg mL^−1^), 3.8 μL of sodium hydroxide (1 M) and 19.5 μL of EDTA (0.17 M) in order on ice. After vortexing for 5 min, the gel was carefully applied to a microslide capillary tube (Vitrocom no. 2540) and incubated at 37 ℃ for 12 h. Then, 10 μL of FITC-labeled MPNs solutions (1.0 mg mL^−1^) after incubation at pH 7.4 or pH 6.5 for 1 h were added into the capillary tube and incubated at 37 ℃ for another 12 h. A CLSM (Leica TCP SP5) was used to capture the images. And the images were analyzed using a software Image J.

### In vitro drug penetration

Multicellular tumor spheroids from 4T1 cells were prepared by the method as previously reported^38^. Briefly, 2 × 10^5^ of 4T1 cells in 2 mL of RMPI 1640 medium were seeded into 6-well plates whose bases coated with 2 mL of 1% agarose per well. When reached about 200 μm in diameter, these MTSs were transferred to a new glass-bottomed dishes and treated with DOX, D-NPs or D-MPNs in the pH 7.4 or 6.5 culture media at a DOX concentration of 5 μg mL^−1^ for 2 h. The MTSs were then gently washed with PBS 3 times and viewed with a CLSM. For quantification, the MTSs treated with DOX formulations were dissociated into single cells by accutase regent (Invitrogen, USA) and the DOX fluorescence was detected with flow cytometry.

### In vitro cytotoxicity

The cytotoxicity of D-MPNs to 4T1 cells was evaluated by CCK-8 assays. The cells were cultured in 96-well plates at a density of 5 × 10^3^ cells per well for 24 h, followed by treatment with DOX∙HCl, DOX, D-NPs or D-MPNs at different drug concentrations from 0.001 to 100 μg mL^−1^ for another 48 h. After rinsing with PBS 3 times, the cells were incubated with 100 μL of FBS-free medium containing 10% (v/v) CCK-8 for additional 2 h. The absorbance data at 450 nm were measured by a Varioskan Flash microplate reader (Thermo Fisher Scientific, USA). And the cell viabilities were determined according to the following formula: cell viability (%) = (OD_sample_—OD_background_)/(OD_control_—OD_background_) × 100%.

To elucidate the influence of intracellular GSH on the cytotoxicity of D-MPNs, cells were pretreated with GSH-OEt (10 mM) for 2 h. After exposure to D-MPNs at a DOX concentration ranging from 0.05 to 5 μg mL^−1^ for 48 h, the cell viability was determined by CCK-8 assays.

### In vivo antitumor effect

When the tumor volumes reached about 250 mm^3^, the mice were randomly assigned into 4 groups (n = 8). Animals were intravenously administrated with saline, MPNs, DOX·HCl, D-NPs or D-MPNs every 3 days for 4 times (injection dose: 5 mg DOX/kg body mass). The tumor volume (*V*) was calculated from the equation: *V* (mm^3^) = *l·w*^2^/2, where *l* and *w* were the length and width of tumor. The relative body weight (w/w_0_) was calculated by the equation: w/w_0_ = (body weight) /(body weight when the treatment was initiated).

### Histological and immunohistochemical analyses

At the end of the treatment course, solid tumors and major organs were excised, fixed in 4% (v/v) formalin solution and made of paraffin sections. These sections were further stained with terminal deoxynucleotidyl transferase mediated UTP end labeling (TUNEL), platelet endothelial cell adhesion molecules-1 (CD31), nuclear nonhistone protein Ki67 and hematoxylin and eosin (H&E). Then the tissue slices were observed with an inverted fluorescence microscope (Leica DMI 4000B, Germany).

### In vivo biodistribution

4T1 tumor-bearing BALB/c mice were administrated with DOX·HCl, D-NPs or D-MPNs suspension (10 mg kg^−1^ DOX). At 12 h post-administration, the major organs and tumors were collected and weighed. The tissues were homogenized in a KH_2_PO_4_ solution (10 mL g^−1^ tissue), and then DOX was extracted by chloroform and isopropanol (v/v, 4:1). After centrifugation at 10,000 rpm, the organic layer was transferred to a clean tube to evaporate under a gentle stream of nitrogen. The residue was dissolved in acetonitrile for HPLC analysis.

### Tumor penetration in vivo

The mice bearing 4T1 tumors were intravenously administrated with DOX·HCl, D-NPs or D-MPNs at a 10 mg kg^−1^ dose of DOX. After 12 h, the tumors were separated from mice for frozen sections at a thickness of 8 μm on an equatorial plane. The sections were fixed in acetone for 10 min, blocked in a blocking buffer (PBS with 5% BSA) for 1 h and incubated with FITC-CD31 antibody for 1.5 h. After washed with PBS 3 times, the samples were stained with Hoechst 33,342 and observed under a CLSM. To demonstrate the acid-triggered deep penetration of nanoparticles, a tumor glycolysis inhibitor 2-DG was intratumorally injected (250 mg kg^−1^) 12 h before administration of D-MPNs.

### Pharmacokinetic studies

BALB/c mice (female, ~ 20 g) were administrated with DOX·HCl, D-NPs or D-MPNs via their tail veins at a 10 mg kg^−1^ dose of DOX. After a predetermined time, blood samples were collected via eyeball extirpating into heparinized tubes and centrifuged at 3000 rpm to obtain the plasma. 100 μL of plasma was mixed with 50 μL of 5 M HCl and kept at 50 ℃ for 1.5 h. Followed by mixed with 50 μL of 1 M NaOH, the mixture was extracted with chloroform/isopropanol (v/v, 4:1). After centrifugation (10,000×g, 5 min), the organic phase was transferred to a new tube, evaporated under vacuum, and re-dissolved in 100 μL of mobile phase, acetonitrile: water (v/v, 7:3) with 0.1% (v/v) TFA for HPLC analysis.

## Supplementary Information

**Additional file 1**: **Fig. S1**. ^1^H NMR spectrum (400 MHz) of mPEG-LA conjugates in CDCl_3_. **Fig. S2**. SEM image (A), size (B) and zeta potential (C) of the self-assembly of mPEG-LA conjugates (NPs, 100 g mL^−1^). **Fig. S3**. Size distribution of MPNs at different concentrations. **Fig. S4**. Fluorescence spectra of a mixed solution of NPs (100 μg mL^−1^)and MPNs (100 μg mL^−1^) prepared with 0.1 wt% DiO/DiI tracing the development of FRET between two dyes. **Fig. S5**. Critical aggregation concentration of MPNs at (A) pH 7.4 or (B) 6.5. **Fig. S6**. Fluorescence spectra of a mixed solution of MPNs (100 μg mL^−1^, pH 6.5) prepared with 0.1 wt% DiO/DiI tracing the development of FRET between two dyes over time. **Fig. S7**. Flow cytometric histogram profiles of 4T1 cells treated with MPNs, DOX, D-NPs and D-MPNs for 3 h (DOX concentration: 10 μg mL^−1^). **Fig. S8**. CLSM images of 4T1 cells incubated with DOX, D-NPs, D-MPNs and DOX·HCl for 1 h (DOX dosage: 2 μg mL^−1^). Hoechst 33342 was used to stain the cell nuclei. Scale bar: 10 μm. **Fig. S9**. Quantitative assessment of penetration of DOX formulations into 4T1 cells. The cells were incubated with DOX, D-NPs and D-MPNs at a DOX concentration of 5 μg mL^−1^ for 2 h. **Fig. S10**. IOD (Integrated optical density) of immunohistochemical images for saline, MPNs, DOX·HCl, D-NPs and D-MPNs groups. The apoptotic rates of tumor sections based on TUNEL images (A), tumor microvessel density based on CD31 images (B) and Ki67 positive cells (C) of tumor sections were calculated with Image-Pro Plus 6.0 software. Data were presented as mean ± SD (n = 3), *p < 0.01, **p < 0.005. **Fig. S11**. Body weight changes of 4T1-bearing BALB/c mice after administration with saline, MPNs, DOX·HCl, D-NPs and D-MPNs (n = 8, dosage: 5 mg DOX kg^−1^ mouse body weight, *p < 0.01). The arrows indicated the time points for intravenous injection. **Fig. S12**. Routine blood analysis results of the mice collected on the 12th day after intravenous injection of saline, MPNs, DOX·HCl, D-NPs or D-MPNs. The results show mean and standard deviation of white blood cells (WBCs), red blood cell (RBC), hemoglobin (HGB) and platelets (PLT). **Fig. S13**. Histological examination of major organs separated from 4T1-bearing BALB/c mice after administration with saline, MPNs, DOX·HCl, D-NPs and D-MPNs for 18 days. Scale bar: 100 μm. **Fig. S14**. TUNEL staining of heart (×200) of 4T1-bearing BALB/c mice after administration with saline, MPNs, DOX·HCl, D-NPs and D-MPNs for 18 days. The apoptotic cells (red arrow) and normal cells were stained brown and blue, respectively. **Table S1**. Pharmacokinetic parameters of DOX·HCl, D-NPs and D-MPNs after intravenous administration at an equivalent dose of 10 mg DOX/kg mouse body weight (n = 3 per group).

## Data Availability

All data generated or analyzed during this study are included in this article and its Additional file.
