# Supplementary material for: Facile fabrication of multi-pocket nanoparticles with stepwise size transition for promoting deep penetration and tumor targeting
Source: J Nanobiotechnology. 2021 Apr 19;19:111. doi: 10.1186/s12951-021-00854-z (PMC8054436; doi:10.1186/s12951-021-00854-z)
Supplement: Supplementary file 1 — Additional file 1: Fig. S1. 1H NMR spectrum (400 MHz) of mPEG-LA conjugates in CDCl3. Fig. S2. SEM image (A), size (B) and zeta potential (C) of the self-assembly of mPEG-LA conjugates (NPs, 100 g mL−1). Fig. S3. Size distribution of MPNs at different concentrations. Fig. S4. Fluorescence spectra of a mixed solution of NPs (100 μg mL−1)and MPNs (100 μg mL−1) prepared with 0.1 wt% DiO/DiI tracing the development of FRET between two dyes. Fig. S5. Critical aggregation concentration of MPNs at (A) pH 7.4 or (B) 6.5. Fig. S6. Fluorescence spectra of a mixed solution of MPNs (100 μg mL−1, pH 6.5) prepared with 0.1 wt% DiO/DiI tracing the development of FRET between two dyes over time. Fig. S7. Flow cytometric histogram profiles of 4T1 cells treated with MPNs, DOX, D-NPs and D-MPNs for 3 h (DOX concentration: 10 μg mL−1). Fig. S8. CLSM images of 4T1 cells incubated with DOX, D-NPs, D-MPNs and DOX·HCl for 1 h (DOX dosage: 2 μg mL−1). Hoechst 33342 was used to stain the cell nuclei. Scale bar: 10 μm. Fig. S9. Quantitative assessment of penetration of DOX formulations into 4T1 cells. The cells were incubated with DOX, D-NPs and D-MPNs at a DOX concentration of 5 μg mL−1 for 2 h. Fig. S10. IOD (Integrated optical density) of immunohistochemical images for saline, MPNs, DOX·HCl, D-NPs and D-MPNs groups. The apoptotic rates of tumor sections based on TUNEL images (A), tumor microvessel density based on CD31 images (B) and Ki67 positive cells (C) of tumor sections were calculated with Image-Pro Plus 6.0 software. Data were presented as mean ± SD (n = 3), *p < 0.01, **p < 0.005. Fig. S11. Body weight changes of 4T1-bearing BALB/c mice after administration with saline, MPNs, DOX·HCl, D-NPs and D-MPNs (n = 8, dosage: 5 mg DOX kg−1 mouse body weight, *p < 0.01). The arrows indicated the time points for intravenous injection. Fig. S12. Routine blood analysis results of the mice collected on the 12th day after intravenous injection of saline, MPNs, DOX·HCl, D-NPs or D-MPNs. The result [file 12951_2021_854_MOESM1_ESM.docx]

**Supporting Information**

Facile Fabrication of Multi-pocket Nanoparticles with Stepwise Size Transition for Promoting Deep Penetration and Tumor Targeting

Xingyu Hou,^a^ Dan Zhong,^a^ Yunkun Li,^a^ Hongli Mao,^b^ Jun Yang,^c^ Hu Zhang,^d^ Kui Luo,^a^ Qiyong Gong ^a^ and Zhongwei Gu*^,a,b^

^a^ Huaxi MR Research Center (HMRRC), Department of Radiology, Functional and Molecular Imaging Key Laboratory of Sichuan Province, National Clinical Research Center for Geriatrics, West China Hospital, Sichuan University, Chengdu 610041, P. R. China

E-mail: [zwgu@scu.edu.cn](mailto:zwgu@scu.edu.cn), zwgu@njtech.edu.cn

^b^ Research Institute for Biomaterials, Tech Institute for Advanced Materials, College of Materials Science and Engineering, NJTech-BARTY Joint Research Center for Innovative Medical Technology, Nanjing Tech University, Nanjing 211816, P. R. China

^c^ The Key Laboratory of Bioactive Materials, Ministry of Education, College of Life Science, Nankai University, Tianjin 300071, P. R. China

^d^ Amgen Bioprocessing Centre, Keck Graduate Institute, Claremont, CA 91711, USA

**1 Experimental Section**

**1.1 Materials**

Methoxypolyethylene glycol (mPEG, *M*w = 1000, Aladdin) and lipoic acid (LA, Aladdin), *N*-(3-dimethylaminopropyl)-*N’*-ethylcarbodiimide hydrochloride (EDC∙HCl, GL Biochem), 1-hydroxybenzotriazole hydrate (HOBT, GL Biochem), *N*,*N*-diisopropylethylamine (DIPEA, Asta Tech Pharmaceutical), Dithiothreitol (DTT, Sigma-Aldrich), glutathione (GSH, Sigma-Aldrich), glutathione monoester (GSH-OEt, Sigma-Aldrich) and pyrene (Sigma-Aldrich) were used as received. Doxorubicin hydrochloride (DOX∙HCl) was purchased from Zhejiang Hisun Pharmaceutical Co. and was deprotonated at pH 9.6 to obtain hydrophobic DOX. Cell counting kit-8 (CCK-8) and Hoechst 33342 were obtained from Dojindo Laboratories (Kumamoto, Japan). 1,1'-dioctadecyl-3,3,3',3'-tetramethylindocarbocyanine (DiI), 3,3'-dioctadecyloxacarbocyanine perchlorate (DiO) and Dulbecco's phosphate-buffered saline (D-PBS) were purchased from Beyotime Institute of Biotechnology (Shanghai, China). StemPro® Accutase® cell dissociation reagent was obtained from Life Technologies. BALB/c mice were purchased from Chengdu Dashuo Experimental Animal Company (Chengdu, China). All organic solvents were purified according to standard procedures.

**1.2 Methods**

The products were characterized by ^1^H NMR spectrometer (400 MHz, Bruker Avance II, Germany) and matrix-assisted laser desorption time-of-flight (MALDI-TOF) mass spectrometer (Bruker Autoflex III, Germany). The size distribution and zeta potential of the Nanoparticles (NPs) in water were measured by dynamic light scattering (DLS, Malvern NanoZS90, UK) at 25 ℃. The nanostructure was determined from a scanning electron microscope (SEM, S-4800, HITSCHI, Japan). The differential scanning calorimetry (DSC) curves were obtained by a TA 2000 instrument (USA) from -5 ℃ to 60 ℃ at a heating rate of 10 ℃ min^-1^ in N_2_ atmosphere. The morphology of MPNs at pH 7.4 and 6.5 was also observed under a transmission electron microscope (Tecnai G2 F20 S-TWIN, USA). The samples for were negatively stained with phosphotungstic acid (2 wt %). For gel permeation chromatography (GPC) analysis, the molecular weight of MPNs at pH 7.4 and 6.5 was determined *via* a Waters HPLC system equipped with a model 1515 isocratic pump, a 717 plus autosampler, and a 2414 refractive index (RI) detector with Waters Ultrahydrogel 120 and 250 columns in series. The flow rate was 1.0 mL min^-1^. The sample concentration was 5 mg mL^-1^.

**1.3 Cell Culture** **and Animal Model**

Mouse mammary carcinoma 4T1 cells were cultured in RMPI 1640 media supplemented with 10% fetal bovine serum (FBS) and 1% antibiotics (100 IU mL^-1^ of penicillin and 100 μg mL^-1^ of streptomycin) in a humidified 5% CO_2_ incubator at 37 ℃. BALB/c mice (female, 18 ~ 22 g) were purchased from Chengdu Dashuo Laboratory Animal Technology Co.. To develop a tumor model, 1 × 10^6^ A549/T cells were subcutaneously inoculated into the right flank. When the tumor volume reached about 250 mm^3^, the mice were used for *in vivo* experiments. All animal procedures were performed in accordance with the Guidelines for Care and Use of Laboratory Animals of Sichuan University (Chengdu, China) and the experiments were approved by the ethics committee of Sichuan University (Chengdu, China).

**1.4 *In vivo* toxicity studies**

To evaluate the systemic toxicity of D-MPNs, the mice were intravenously injected with saline, MPNs, DOX·HCl, D-NPs or D-MPNs every 3 days for 4 times. Then, 3 days after the last administration, the mice were sacrificed to harvest the blood for routine blood analysis.

**2 Supplementary Data**





***Fig. S1.*** ^1^H NMR spectrum (400 MHz) of mPEG-LA conjugates in CDCl_3_.


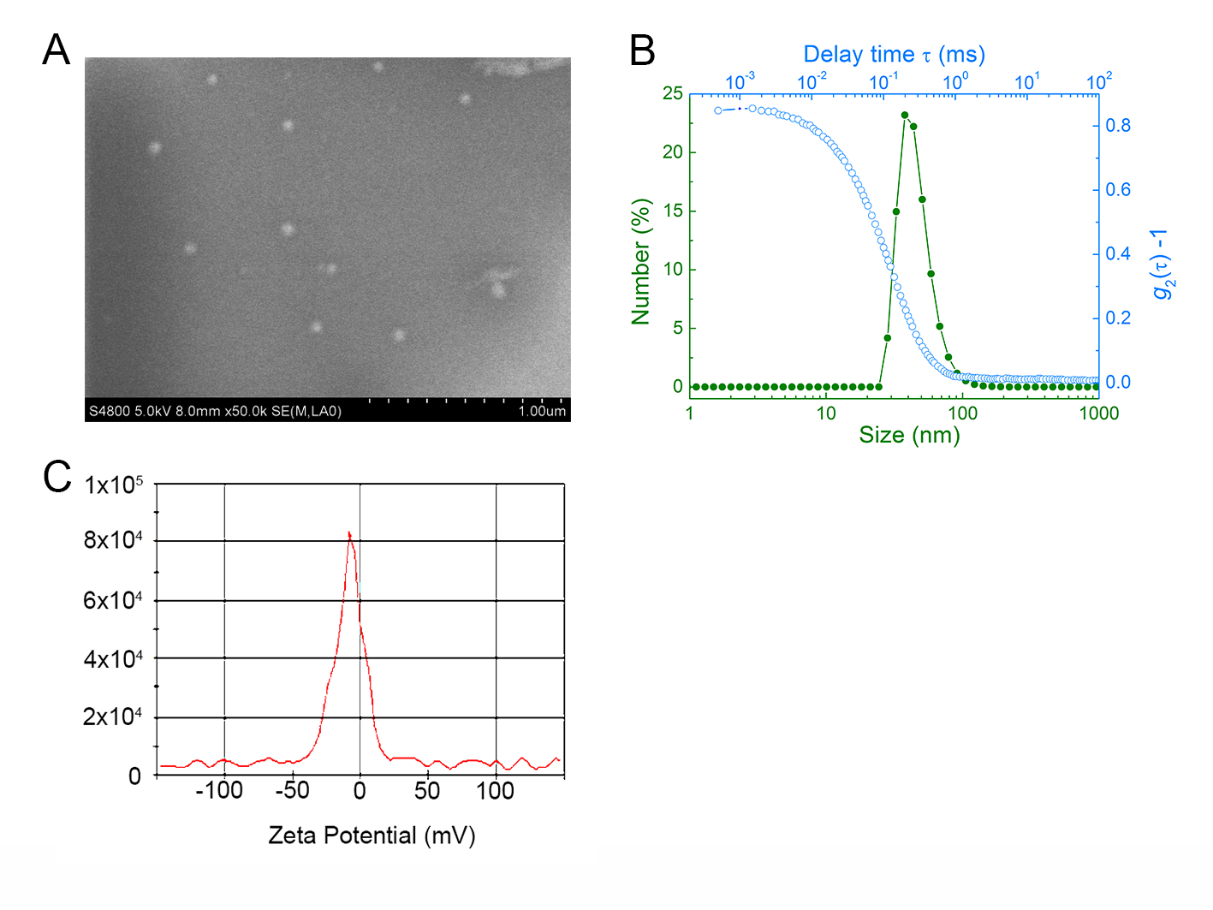


***Fig. S2.*** SEM image (A), size (B) and zeta potential (C) of the self-assembly of mPEG-LA conjugates (NPs, 100 μg mL^-1^).





***Fig. S3.*** Size distribution of MPNs at different concentrations.


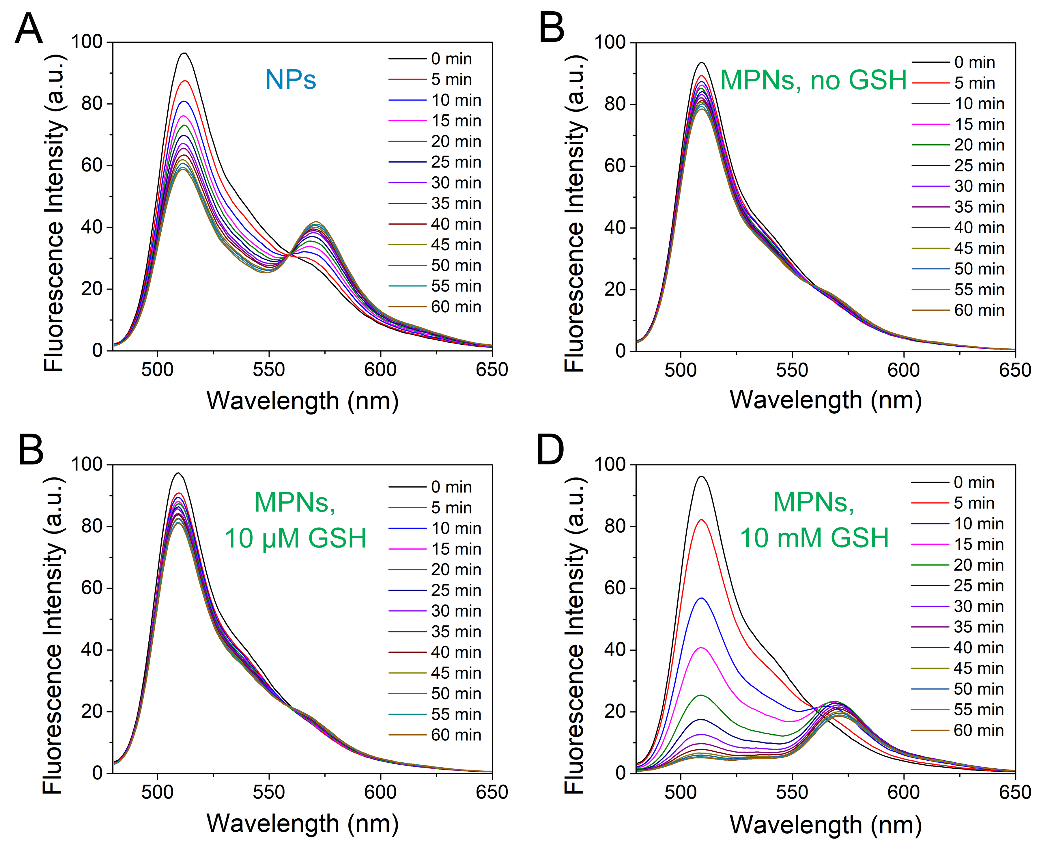


***Fig. S4***. Fluorescence spectra of a mixed solution of NPs (100 μg mL^-1^)and MPNs (100 μg mL^-1^) prepared with 0.1 wt% DiO/DiI tracing the development of FRET between two dyes.


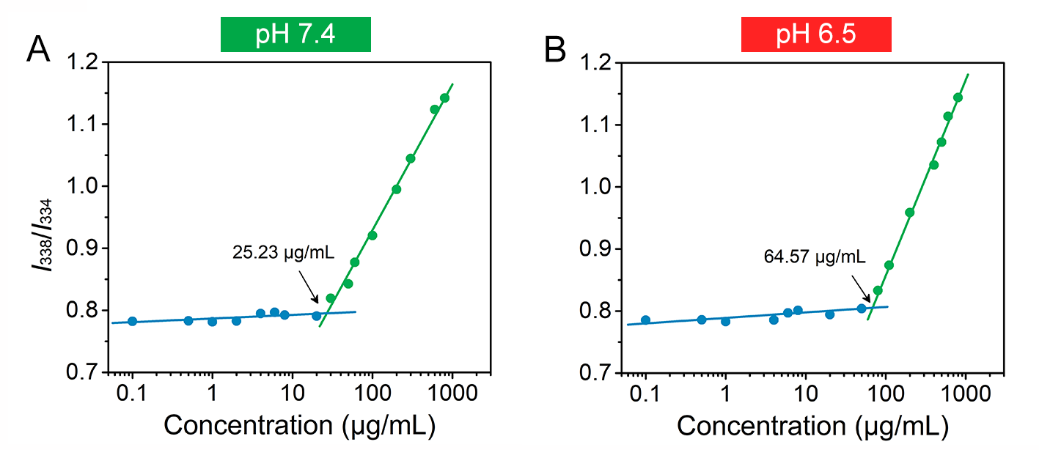


***Fig. S5***. Critical aggregation concentration of MPNs at (A) pH 7.4 or (B) 6.5.





***Fig. S6.*** Fluorescence spectra of a mixed solution of MPNs (100 μg mL^-1^, pH 6.5) prepared with 0.1 wt% DiO/DiI tracing the development of FRET between two dyes over time.


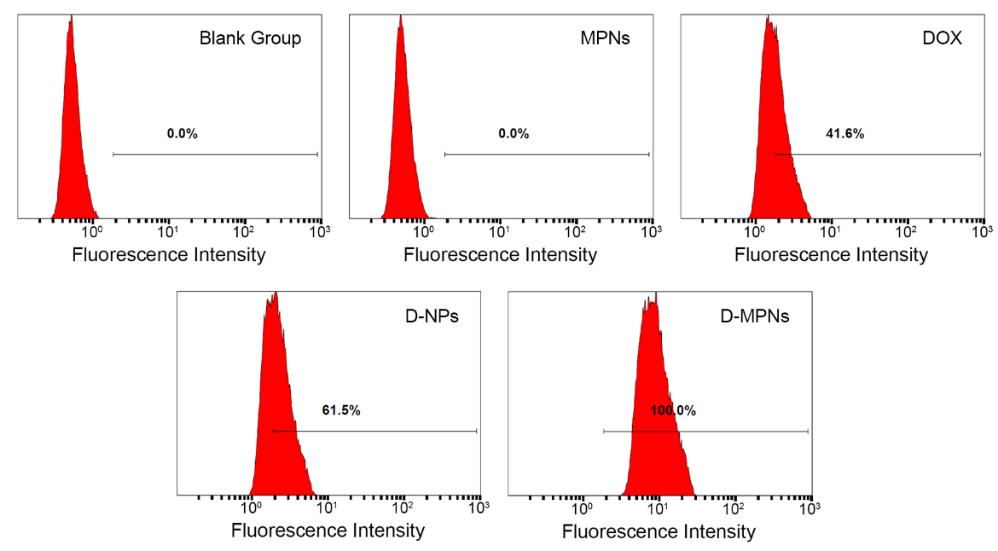


***Fig. S7.*** Flow cytometric histogram profiles of 4T1 cells treated with MPNs, DOX, D-NPs and D-MPNs for 3 h (DOX concentration: 10 μg mL^-1^).


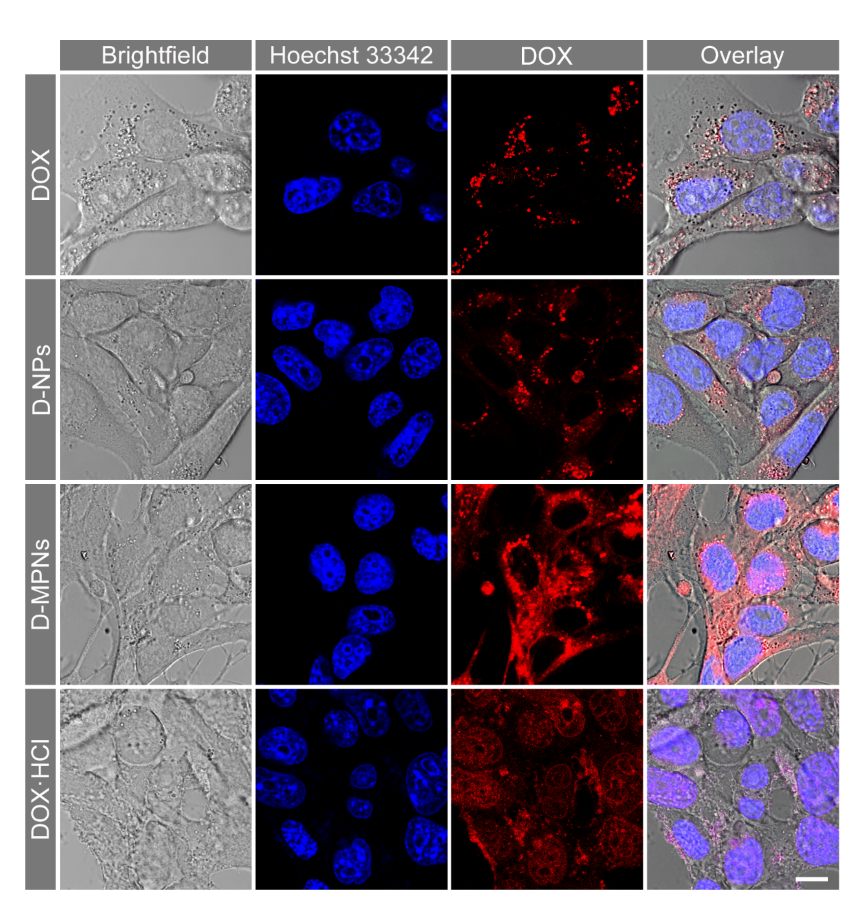


***Fig. S8.*** CLSM images of 4T1 cells incubated with DOX, D-NPs, D-MPNs and DOX·HCl for 1 h (DOX dosage: 2 μg mL^-1^). Hoechst 33342 was used to stain the cell nuclei. Scale bar: 10 μm.


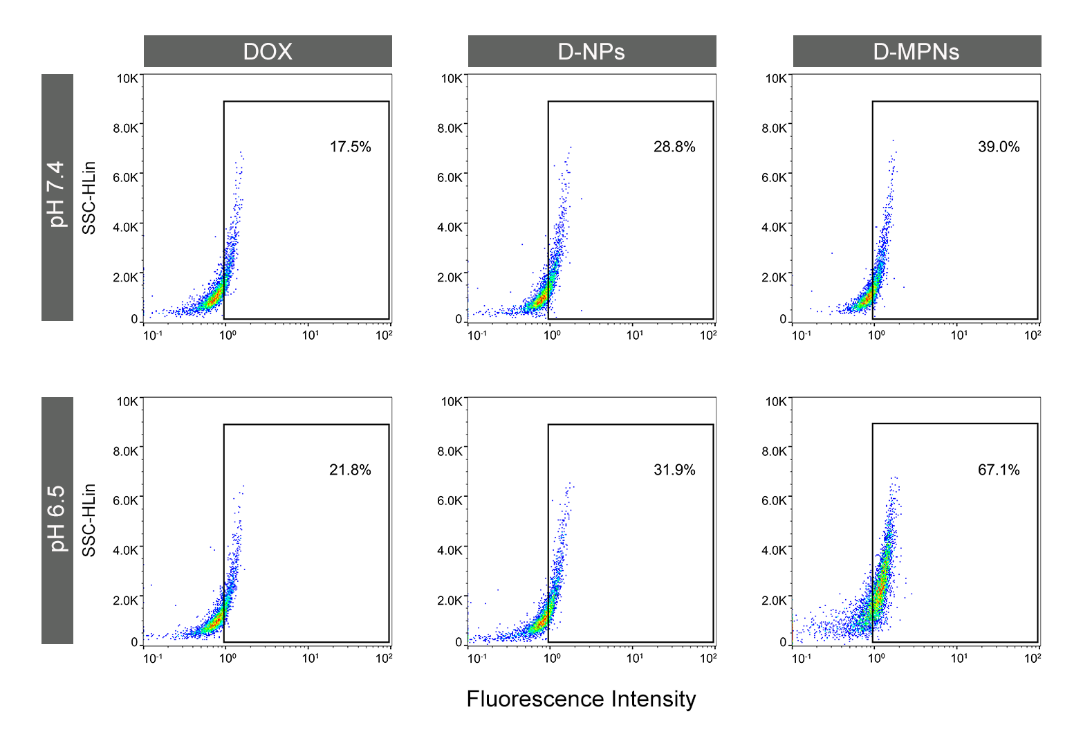


***Fig. S9.*** Quantitative assessment of penetration of DOX formulations into 4T1 cells. The cells were incubated with DOX, D-NPs and D-MPNs at a DOX concentration of 5 μg mL^-1^ for 2 h.


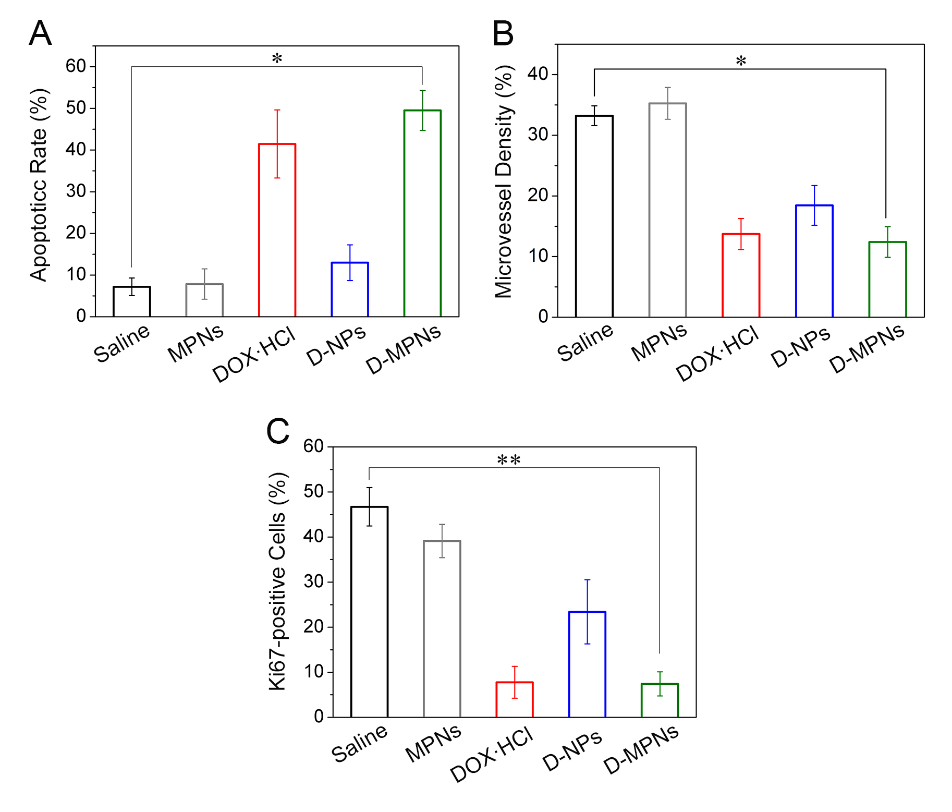


***Fig. S10.*** IOD (Integrated optical density) of immunohistochemical images for saline, MPNs, DOX·HCl, D-NPs and D-MPNs groups. The apoptotic rates of tumor sections based on TUNEL images (A), tumor microvessel density based on CD31 images (B) and Ki67 positive cells (C) of tumor sections were calculated with Image-Pro Plus 6.0 software. Data were presented as mean ± SD (*n* = 3), **p* < 0.01, ***p* < 0.005.





***Fig. S11.*** Body weight changes of 4T1-bearing BALB/c mice after administration with saline, MPNs, DOX·HCl, D-NPs and D-MPNs (*n* = 8, dosage: 5 mg DOX kg^-1^ mouse body weight, **p* < 0.01). The arrows indicated the time points for intravenous injection.


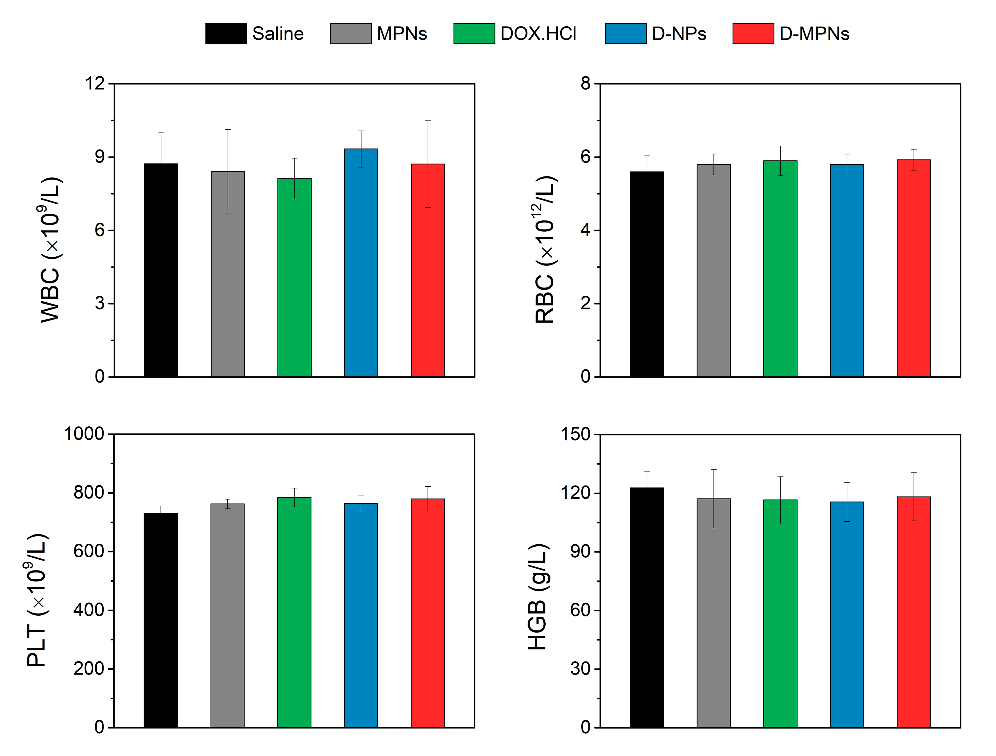


***Fig. S12.*** Routine blood analysis results of the mice collected on the 12th day after intravenous injection of saline, MPNs, DOX·HCl, D-NPs or D-MPNs. The results show mean and standard deviation of white blood cells (WBCs), red blood cell (RBC), hemoglobin (HGB) and platelets (PLT).


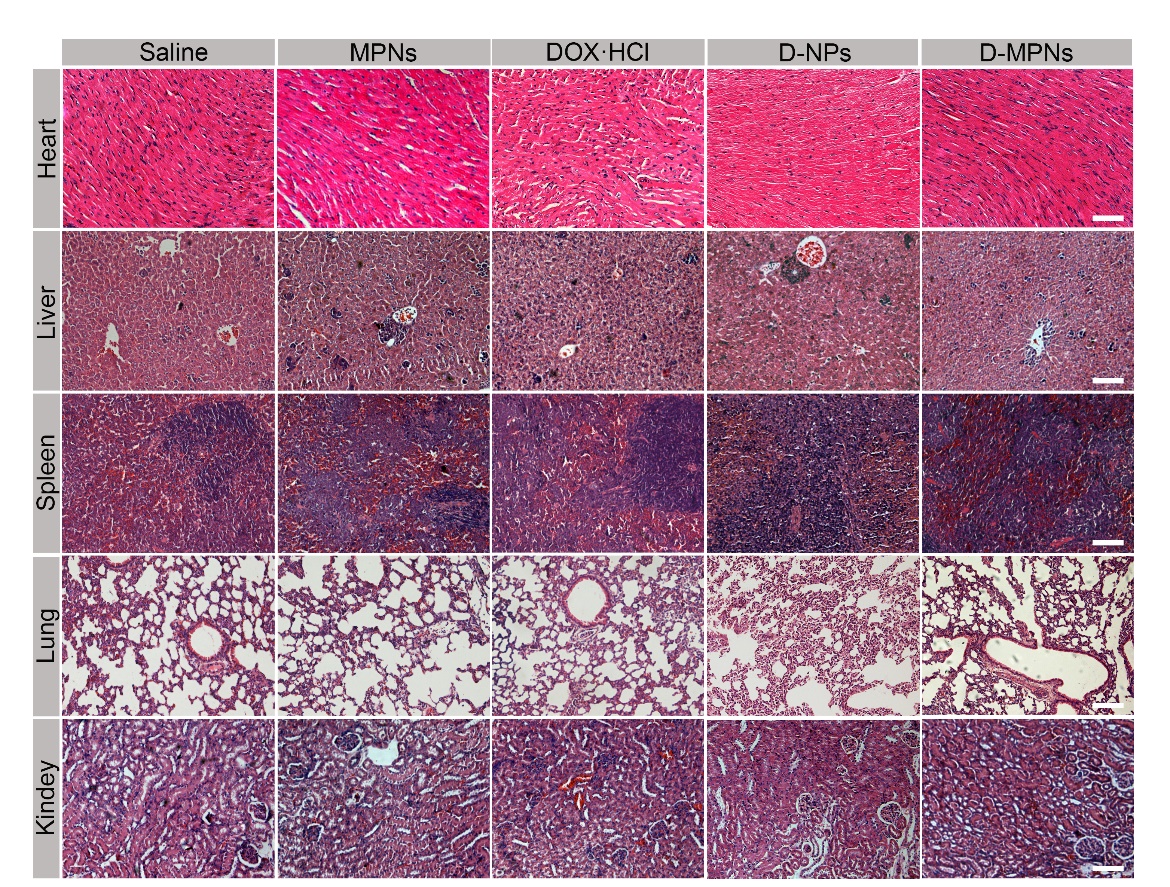


***Fig. S13.*** Histological examination of major organs separated from 4T1-bearing BALB/c mice after administration with saline, MPNs, DOX·HCl, D-NPs and D-MPNs for 18 days. Scale bar: 100 μm.


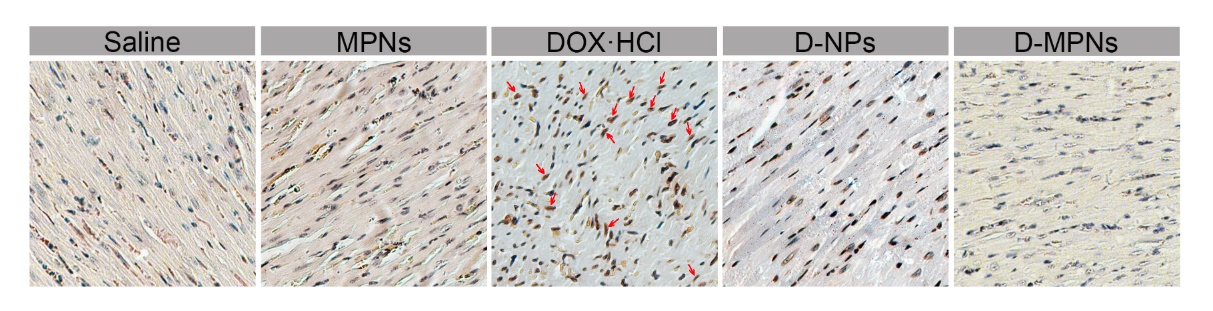


***Fig. S14.*** TUNEL staining of heart (×200) of 4T1-bearing BALB/c mice after administration with saline, MPNs, DOX·HCl, D-NPs and D-MPNs for 18 days. The apoptotic cells (red arrow) and normal cells were stained brown and blue, respectively.

**Table S1.** Pharmacokinetic parameters of DOX·HCl, D-NPs and D-MPNs after intravenous administration at an equivalent dose of 10 mg DOX/kg mouse body weight (*n* = 3 per group).

| Parameter | DOX·HCl | D-NPs | D-MPNs |
| --- | --- | --- | --- |
| MRT_0-∞_ ^a^ (h) | 0.49 | 3.04 | 39.44 |
| t_1/2z_ ^b^ (h) | 0.83 | 1.81 | 18.09 |
| AUC_0-∞_ ^c^ (μg mL^-1^×h) | 59.54 | 102.85 | 641.01 |

^a^ Mean retention time.

^b^ Elimination half-life.

^c^ Area under curve.
